# Anti-*Campylobacter* Activity of Ternary Copper(II) Complexes with Imine Ligands and 4′-(4-Methylphenyl)-2,2′:6′,2″-Terpyridine

**DOI:** 10.1021/acsomega.5c06248

**Published:** 2026-01-23

**Authors:** Micaela G. Takeuchi, Ana Laura M. Ferreira, Luana M. S. Ramos, Jéssica Laura M. Peixoto, Mariana C. Chueiri, Carolyne F. Dumont, Gabriella R. A. Ferreira, Diogo M. de Jesus, Thiago dos S. Ramos, André L. Bogado, Gabriele de M. Pereira, Marcelo C. Portes, Pedro P. Corbi, Ana Maria da C. Ferreira, Daise A. Rossi, Wendell Guerra, Roberta T. de Melo

**Affiliations:** † Federal Institute of Rio Grande do Sul, Farroupilha Campus, Farroupilha, Rio Grande do Sul 95174-274, Brazil; ‡ Molecular Epidemiology Laboratory, Federal University of Uberlândia, Umuarama Campus, Uberlândia, Minas Gerais 38400-902, Brazil; § Institute of Chemistry, Federal University of Uberlândia, Santa Mônica Campus, Uberlândia, Minas Gerais 38400-902, Brazil; ∥ Department of Fundamental Chemistry, Institute of Chemistry, University of São Paulo, São Paulo, São Paulo 05508-000, Brazil; ⊥ Department of Chemistry, Institute of Exact and Natural Sciences of Pontal, Federal University of Uberlândia, Ituiutaba, Minas Gerais 38304-402, Brazil; # Institute of Chemistry, 28132 State University of Campinas—UNICAMP, Campinas, São Paulo 13083-862, Brazil

## Abstract

Herein, two Cu­(II) complexes of the type [Cu­(N–N)­(mftpy)]­(PF_6_)_2_ (N–N = 4-chloro-*N*-(pyridin-2-methylene) aniline (Clmp) or 4-methyl-*N*-(pyridin-2-methylene) aniline (memp)­and mftpy = 4′-(4-methylphenyl)-2,2′:6′,2″-terpyridine) were successfully synthesized and characterized by microanalysis (% CHN), high-resolution mass spectrometry, Fourier-transform infrared spectroscopy, and ultraviolet–visible (solution and solid state) and electron paramagnetic resonance spectroscopies (solution and solid state). Next, the in vitro antibacterial activity of the [Cu­(Clmp)­(mftpy)]­(PF_6_)_2_ **CL1** and [Cu­(memp)­(mftpy)]­(PF_6_)_2_ **CL2** complexes was investigated in the planktonic and sessile form of *Campylobacter jejuni* and *Campylobacter coli* strains selected from a bank of strains characterized by resistance to first-line antibiotics. The quantification of planktonic cells showed a reduction that varied from 1.3 to 6.9 log CFU (colony forming units)/mL at a minimum inhibitory concentration of 25–400 μg/mL according to the tested strain. The biofilms suffered modification in their ultrastructure and showed evidence of the action of both complexes that surpassed the results with peracetic acid, with a reduction ≥2.6 log CFU/mL of sessile *Campylobacter*, with control of 1.2 orders of magnitude in the biomass formation by **CL2**, and the highest penetration (4.92 μg) of **CL1** into the *C. jejuni* biofilm. The results identified show that both complexes are biologically active, activating processes that allow the control of the pathogen in both lifestyles.

## Graphical abstract

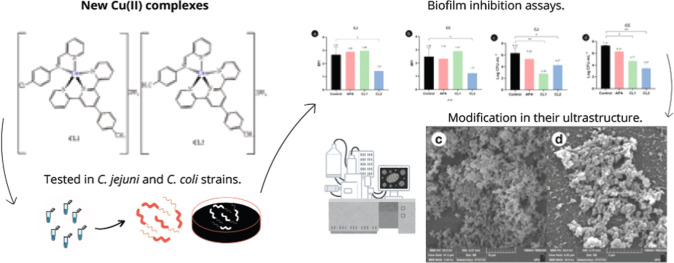

## 1 Introduction

Species of the genus *Campylobacter* are among the leading causes of gastroenteritis worldwide,^1^ and concerns about this pathogen are exacerbated by reports of multiple resistances, especially to first-choice antimicrobial agents such as fluoroquinolones and ciprofloxacin, given high priority by the WHO.^2–5^

Knowledge gaps for *Campylobacter* control^6^ have been investigated mainly related to the development and research of new molecules that can be effective in the treatment of campylobacteriosis or in the environmental control of farm and abattoir level.^7^ Considering that at least 75% of the antimicrobials in the clinical development phase are derived from formulas that have already been used, there is concern about their efficacy against the pathogens’ existing resistance mechanisms, and among the entirely new compounds, only one has obtained satisfactory results in resistant Gram-negatives.^8^

A new generation of effective compounds is critically needed, and efforts to find molecules that are selective for pathogens and do not induce resistance of the microorganisms^9^ contribute to these molecules being candidates to combat this disease.^10^ Copper-based compounds have been studied for their antimicrobial activity^11^ in controlling different microorganisms.^8^ Although it is a crucial micronutrient for physiological processes such as electron transfer activity, oxygen and enzyme cofactor, in excess it can disrupt an intracellular metal homeostasis^12^ which makes it possible to exploit this activity because it is toxic to microbial cells and it can be toxic to them.^13^

The relative contributions of copper ions can be enhanced by the association with different ligands that promote a dual effect on the microorganism, such as Schiff or imine bases, that stabilize metals in different oxidation states and control their performance in useful catalytic transformations. These ligands increase bactericidal activity by decreasing ionic polarity through positive charge sharing with the donor group of the active compound, while increasing the lipophilic nature of the ions and their ability to permeate the lipid layers of the cell membrane, thereby inhibiting bacterial growth.^14^

Regarding the above discussions, our objectives here were to prepare new Cu­(II) complexes bearing imine and 4′-(4-methylphenyl)-2,2′:6′,2″-terpyridine as ligands and evaluate them against *Campylobacter jejuni* and *Campylobacter coli* strains, in planktonic and sessile forms. We consulted the literature, and this is the first work that investigated Cu­(II) complexes with terpyridine or imine ligands against *C. jejuni*.

## 2 Materials and Methods

### 2.1 Reagents and Solvents

The reagents 4′-(4-methylphenyl)-2,2′:6′,2″-terpyridine and Cu­(NO_3_)_2_·3H_2_O were purchased from Merck and were used as received.

### 2.2 Syntheses of the Ligands

The ligands 4-chloro-*N*-(pyridin-2-methylene) aniline (Clmp) and 4-methyl-*N*-(pyridin-2-methylene) aniline (memp) were prepared, as described in the literature,^2^ by the condensation of 2-pyridinecarboxyaldhehyde with para-substituted aniline.

Analytical results for Clmp: yield: 36% (0.39 g). Color: yellow. MM (g mol^–1^): 216.66. RMN ^1^H (400 MHz; CDCl_3_): δ (ppm): 8.74 (d, 1H, ^3^ *J*_H–H_ = 5.1 Hz, H2), 8.60 (s, 1H, H7), 8.21 (d, 1H, ^3^ *J*_H–H_ = 7.4 Hz, H5), 7.85 (t, 1H, H6), 7.40 (m, 3H, H1, H11, H13), 7.25 (s, 2H, H10, H14). IV attenuated total reflectance (ATR), ν (cm^–1^): 1624 (νC=Nimino), 1602 (νC=C and νC=Npyridine), 1584 (νC=C and νC=Npyridine), 1564 (νC=C and νC=Npyridine). Ultraviolet–visible (UV–vis) (ACN), λ_max_ (nm/L mol^–1^ cm^–1^): 323 (7.1 × 10^3^), 282 (1.3 × 10^3^), 232 (1.3 × 10^3^).

Analytical results for memp: yield: 84% (0.83 g). Color: yellow. MM (g mol^–1^): 196.25. RMN ^1^H (400 MHz; CDCl_3_): δ (ppm):8.73 (m, 1H, H2), 8.65 (s, 1H, H7), 8.22 (d, 1H, ^3^ *J*_*H*–H_ 7.9 Hz, H5), 7.83 (m, 1H, H6), 7.39 (m, 1H, ^4^ *J*_H–H_ 1.4 Hz, H1), 7.25 (s, 4H, H10, H11, H13, H14), 2.41 (s, 3H, H15 or CH_3_). IV (ATR), ν (cm^–1^): 1626 (νC=Nimino), 1601 (νC=C and νC=Npyridine), 1583 (νC=C and νC=Npyridine), 1567 (νC=C and νC=Npyridine). UV–vis (ACN), λ_max_ (nm/L mol^–1^ cm^–1^): 333 (6.2 × 10^3^), 283 (9.7 × 10^3^), 234 (1.1 × 10^4^).

### 2.3 Syntheses of the Copper Complexes

The Cu­(II) complexes [Cu­(Clmp)­(mftpy)]­(PF_6_)_2_ (**CL1**) and [Cu­(memp)­(mftpy)]­(PF_6_)_2_ (**CL2**) (Figure 1) were obtained by reacting Cu­(NO_3_)_2_·3H_2_O with mftpy (4′-(4-methylphenyl)-2,2′:6′,2″- terpyridine) and Clmp or memp ligands, depending on the complex, in a molar ratio of 1:1:1 in methanol. The mixture was stirred at room temperature for 24 h. Afterward, 2 mol of NH_4_PF_6_ previously dissolved in water was added to the mixture. Subsequently, the compounds were washed several times with water, filtered off, and dried under reduced pressure.

**Figure 1:**
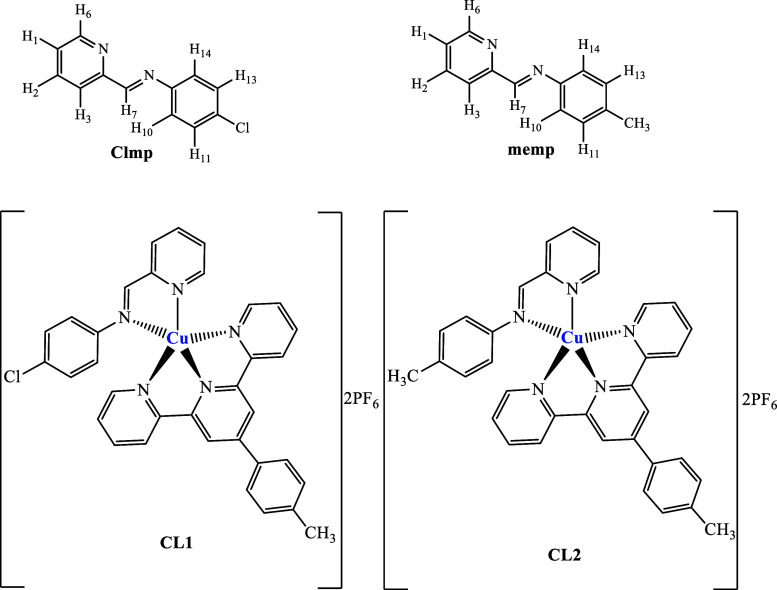
Proposed structures for the imine ligands and the corresponding copper­(II) complexes.

[Cu­(Clmp)­(mftpy)]­(PF_6_)_2_(**CL**_**1**_)/MM (g mol^–1^): 893.53. Yield: 90%. Anal. Calcd for CuC_34_H_26_ClF_12_N_5_P_2_: C, 45.70; H, 2.93; N, 7.84%; Anal. Found: C, 45.80; H, 2.73; N, 7.90%. High-resolution mass spectrometry (HRMS) *m*/*z* (methanol): 301.0586 [M – 2PF_6_]^2+^ (calc. for [CuC_34_H_26_ClN_5_]^2+^, 301.0581 (Δ + 1.6608 ppm)). IV (ATR), ν (cm^–1^): 1620 (νC=Nimino), 1605 (νC=C and νC=Npyridine), 1574 (νC=C and νC=Npyridine), 1558 (νC=C and νC=Npyridine), 827 (νP–F). UV–vis (ACN), λ_max_ (ε): 326 (2.3 × 104 M^–1^ cm^–1^), 286 (3.1 × 104 M^–1^ cm^–1^), 230 (4.4 × 104 M^–1^ cm^–1^), 617 (solid state), 616 (46.4 M^–1^ cm^–1^). ΛM (ACN) = 281.75 S cm^2^ mol^–1^.

[Cu­(memp)­(mftpy)]­(PF_6_)_2_(**CL**_**2**_): MM (g mol^–1^): 915.18. Yield: 75%. Anal. Calcd for CuC_38_H_35_F_12_N_5_P_2_: C, 48.15; H, 3.35; N, 8.02%; Anal. Found: C, 48.20; H, 3.31; N, 8.06%. HRMS *m*/*z* (methanol): 291.0859 [M – 2PF_6_]^2+^ (calc. for [C_35_H_29_CuN_5_]^+2^, 291.0854 (Δ + 1.7177 ppm)). IV (ATR), ν (cm^–1^): 1620 (νC=Nimino), 1605 (νC=C and νC=Npyridine), 1573 (νC=C and νC=Npyridine), 1558 (νC=C and νC=Npyridine), 819 (νP–F). UV–vis (ACN), λ_max_ (ε): 329 (2.9 × 104 M^–1^ cm^–1^), 287 (3.4 × 104 M^–1^ cm^–1^), 228 (4.9 × 104 M^–1^ cm^–1^), 612 (solid state), 612 (45.0 M^–1^ cm^–1^). ΛM (ACN) = 298.38 S cm^2^ mol^–1^.

### 2.4 Strains

We used six strains, including four strains isolated from chicken carcasses by the Brazilian Ministry of Agriculture, Livestock, and Supply (MAPA) and stored in the strain bank at the Federal University of Uberlândia, chosen for their mutual resistance to ciprofloxacin and erythromycin. Two other standard culture strains (ATCC) were evaluated together in determining the minimum inhibitory concentration (MIC). Samples preserved in a cryoprotectant supplemented with ultra-high-temperature milk were revived on Campylobacter Agar Base Blood Free (CCDA) (Oxoid) and maintained in microaerophilic medium (Probac) at 37 °C for 48 h.^15^ The morphological evaluation of typical colonies by Gram staining confirmed the presence of curved Gram-negative bacilli.

### 2.5 Minimum Inhibitory Concentration

The determination of MIC for metal compounds was established against free forms of strains tested in the study using the microdilution method.^16^ Adjusted Mueller–Hinton (MH) broth (Oxoid) was supplemented with Ca^2+^, Mg^2+^, and 5% defibrinated sheep blood (Laborclin). The same medium was used for testing after adding the stock solution (400 μg/mL) and the bacterial suspension prepared in sterile 0.85% NaCl. The bacterial inoculum was adjusted at a concentration corresponding to 0.5 on the McFarland scale and copper complex concentrations ranging from 400 to 3125 μg/mL. Subsequently, the bacterial suspension was inoculated into microplates and incubated at 37 °C for 48 h under microaerophilic conditions. The MIC value was visually determined as the lowest concentration that shows no turbidity, evidenced by a change in medium coloration. Negative controls containing only the medium without bacterial inoculation were included in all assays.

### 2.6 Biofilm Inhibition Formation

To evaluate the inhibition of biofilm formation, qualitative and quantitative analyses of the sessile structure were performed using wild strains (two of *Campylobacter jejuni* (CJ) and two of *C. coli* (CC)). In the quantitative analysis, the bacterial suspension (10^4^ colony-forming units (CFU)/mL; OD600 = 0.22–0.28) was centrifuged at 5000 rpm for 10 min at 4 °C. The pellet obtained was washed twice with sterile 0.9% NaCl solution, after which 20 mL of MH broth (Merck) was added, plus 800 μg/mL of respective reagents (control group, peracetic acid, **CL1,** and **CL2**) and supplemented with 5% chicken juice (cj) to mimic the industry conditions.^17^

Qualitative biofilm staining was performed as previously recommended,^18^ with modifications in eight repetitions on three occasions. In a nutshell, 200 μL of the bacterial suspension diluted with MH broth or MH broth supplemented with cj was dispensed into 96-well plates. The plates were incubated for 48 h to grow biomass. Following incubation, the wells were washed and dried without destaining, and the whole biomass was fixed with 0.1% crystal violet (LaborClin). The dye retained by the biofilm was solubilized with ethanol/acetone (80:20, v/v; Dinamica). Biofilm formation index (BFI) was calculated in accordance with the method outlined by Stepanovic et al.,^19^ obtained from the reading of OD600.

### 2.7 Bacterial Count in Different Lifestyles

To estimate log_10_ reduction, 100 μL from the maximum concentration (400 μg/mL) was transferred into sterile saline and subjected to serial dilutions (10^–1^ to 10^–8^), which were then used for colony enumeration and log reduction analysis. For the biofilms, the highest concentration (800 μg/mL) was evaluated following the same procedure and compared with the control group and the peracetic acid composite group.

### 2.8 Scanning Electron Microscopy

The ultrastructure of the sessile cells from the control group, peracetic acid, and metallic treatments was analyzed using scanning electron microscopy (SEM) according to a modified protocol. Based on the growth conditions described above, biofilm formation was tested on 5 mm glass beads in MH medium. After incubation with the respective treatments, the samples were fixed overnight at 4 °C in a solution containing 2.5% glutaraldehyde and 2.5% paraformaldehyde in 0.1 M phosphate-buffered saline (PBS) (pH 7.4). Following fixation, the samples were washed three times with PBS buffer. The beads were subsequently postfixed in 1% osmium tetroxide for 1 h and washed three times with PBS. Dehydration was performed in a series of ethanol (30, 40, 50, 60, 70, 80, 90%, and three times at 100%) for 15 min per setup. The samples were then dried using critical point drying (030, Baltec, Germany) with liquid CO_2_ as the transitional fluid, coated with a 20 nm gold layer (SCD 050, Baltec, Germany), and examined with a Zeiss Supra 55 FEG SEM operating at 20 kV.

### 2.9 Apparatus

Percentages of carbon, hydrogen, and nitrogen (CHN) were determined in the samples using a PerkinElmer 2400 elemental analyzer. A Tecnopon mCA-150 conductivity meter was used to measure conductivity using acetonitrile as the solvent. HRMS spectra were measured on an Orbi-trap Thermo Q-Exactive (Thermo Fisher Scientific) spectrometer, operating in positive mode. Samples containing 1.0 mg of Cu­(II) complexes were diluted in 1.00 mL of methanol and then diluted in the proportion of 50 μL to 1.00 mL of methanol. Acetronitrile: water (1:1) with 0.1% of formic acid was used as the solvent system, and the samples were infused into the ESI source at a flow rate of 200 μL/min^–1^. Values for charged complex ions were estimated using the software Chem Draw Ultra 15.0. The UV–vis absorption spectra (200–800 nm) were obtained on a Shimadzu spectrophotometer. Infrared (IR) spectra were obtained on a PerkinElmer Frontier MIR spectrometer equipped with an ATR—sample holder with a diamond crystal in the region 4000–400 cm^–1^. Electron paramagnetic resonance (EPR) spectroscopic measurements were registered on an EMX Bruker instrument (Kalsruhe, Germany), working at the X-band (9.5 GHz, 100 kHz modulation amplitude, and 20 mW power). Samples were introduced in quartz tubes (4 mm internal diameter) and measured in the solid state, or introduced in flat cells as dimethyl sulfoxide (DMSO) solution, at room temperature. 2,2-Diphenyl-1-picrylhydrazyl (DPPH) was used as a frequency calibrant (*g* = 2.0036). EasySpin^20^ in combination with MATLAB 2015a platform (MathWorks) was used to perform the corresponding simulations.

Copper concentrations in ppm were obtained with a SpectrAA-220 flame atomic absorption spectrometer (Varian, Australia) equipped with a deuterium lamp for background correction and a multielement hollow-cathode lamp (Agilent, Australia) for absorbance measurement. Calibration curves were made using standard solutions prepared by dilutions of 1 g/L copper solution (SpecSol, Brazil) in media acidized with HNO_3_ and with optimized parameters, for maximum peak height in absorbance measurements, such as flow rate and burner height. All standard solutions and sample dilutions were made with type 1 ultrapure water, 18.2 MΩ cm (Millipore, USA).

### 2.10 Statistical Analyses

The results of the data organized in tables were then analyzed using descriptive statistics, and normality was tested for both qualitative and quantitative data. Pairwise comparisons were made either by *t* test or Mann–Whitney test, whereas analyses of three or more groups were conducted using analysis of variance (ANOVA) or the Kruskal–Wallis method (95% confidence level). The statistics were performed on GraphPad Prism version 8.0.1.

## 3 Results

### 3.1 Synthesis and Spectroscopic Characterization of Complexes

The Clmp and memp ligands were prepared as described in the literature, and their ^1^H NMR spectra (Figures S1 and S2) confirm their identities and purities. Subsequently, two new copper­(II) complexes, named [Cu­(Clmp)­(mftpy)]­(PF_6_)_2_ (**CL1**) and [Cu­(memp)­(mftpy)]­(PF_6_)_2_ (**CL2**), were obtained and characterized by a set of physical–chemical methods.

As to the chemical structures proposed to these complexes (Figure 1), both are 1:2 electrolytes, with molar conductivity values close to 285 S cm^2^ mol^–1^. Mass spectra (Figures S3 and S4) were measured, and the data are in good agreement with the proposed structures. For instance, the mass spectrum of **C1L** showed a charged complex ion at *m*/*z* 301.0586 [M – 2PF_6_]^2+^, which agrees with the calculated value (301.0581), with a mass error (Δ) of +1.6608 ppm for [CuC_34_H_26_ClN_5_]^2+^.

Regarding the UV–vis spectra (Figures S5 and S6), both ligands showed a band around 232 nm, attributed to the π → π* transition of the imine group and two bands close to 283 and 330 nm assigned to the π → π* transitions of the pyridine and phenyl groups, respectively.^21^ As to the d–d transitions, in the solid state, the complexes exhibited a band close to 614 nm (Figure S7). For example, complex **C2L** showed a d–d band centered at 612 nm. In solution (acetonitrile), this band also appeared at the same position (612 nm, ε = 45 mol^–1^ L cm^–1^). The same behavior was observed for **C1L** (see Experimental Section and Figure S7). The Fourier-transform infrared (FTIR) spectra of the copper­(II) complexes (Figures S8 and S9) corroborate the presence of coordinated ligands to the copper­(II) ion through nitrogen atoms. For instance, the FTIR spectra of the ligands showed C=N bands at around 1625 cm^–1^, whereas in the complexes, these same bands were found to be slightly shifted. Furthermore, the complexes exhibited a broad and strong band near 820 cm^–1^, attributable to the stretching vibrations of the PF_6_^–^ anion.

Figures S10 and S11 show the EPR spectra of **CL1** and **CL2** complexes. They exhibit very close structural features, as expected, indicating a typical square-pyramidal environment around the copper ion, with similar spectroscopic parameters, as displayed in Table 1. The tridentate ligand (mftpy) is in the equatorial plane, while the bidentate imine ligand occupies the fourth equatorial position and an axial site. Simulation calculations indicated low values for *A*_⊥_ and *A*_//_, consistent with the experimental results. The hyperfine structures observed at the *g*_⊥_ signal (up to 7 lines) are indicative of the five nitrogen atoms coordinated to copper in both complexes.

**Table 1:** EPR Parameters of Copper­(II) Complexes **CL1** and **CL2**, in Solid State and in DMSO Solution, at Room Temperature

complex	*g*_⊥_	*g*_//_	*A*_⊥_, *G*	*A*_//_ (10^–4^ cm^–1^)
**CL1**	2.0420	2.2050		16 G
solid state				
DMSO solution	2.0756	2.1586	3.4	17 G
**CL2**	2.0526	2.2150		16 G
solid state				
DMSO solution	2.0729	2.1486	7.0	13 G

### 3.2 Anti-*Campylobacter* Activity

We found that the strains tested behaved differently toward the complexes, even when they were the same species. The ATCC strains were more susceptible to both complexes. The MIC results are described in Table 2.

**Table 2:** Anti-*Campylobacter* Activity (MIC) of Complexes **CL1** and **CL2**

strain	CL1 (μg/mL)	CL2 (μg/mL)
CJ ATCC	25	50
CJ 143	400	200
CJ 68/7	400	400
CC ATCC	50	50
CC 78/2	400	200
CC 60/7	400	400

The comparative analysis between the capability of reducing the initial concentration (6.98 log CFU/mL) at 400 μg/mL showed a difference in behavior between CJ and CC, such that CJ68 showed the greatest susceptibility, with growth of less than 10 CFU/mL (equivalent to a reduction of 99.9999% of the bacteria), and CC78/2 the greatest resistance, with an average reduction of 1.13 log CFU/mL, for both compounds.

Overall, we observed that **CL1** and **CL2** promoted a reduction of 3.19 and 3.68 log CFU of bacteria compared to the control, respectively (*p* < 0.0001—Kruskal–Wallis test), with no difference between the complexes (*p* = 0.9719—Mann–Whitney test) (Figure 2). However, the complex associated with the Clmp ligand (**CL1**) was more effective in reducing the bacterial concentration of CJ ATCC when compared to the complex associated with the memp ligand (**CL2**). For the wild strains, **CL1** and **CL2** exhibited efficacy at higher concentrations (200–400 μg).

**Figure 2:**
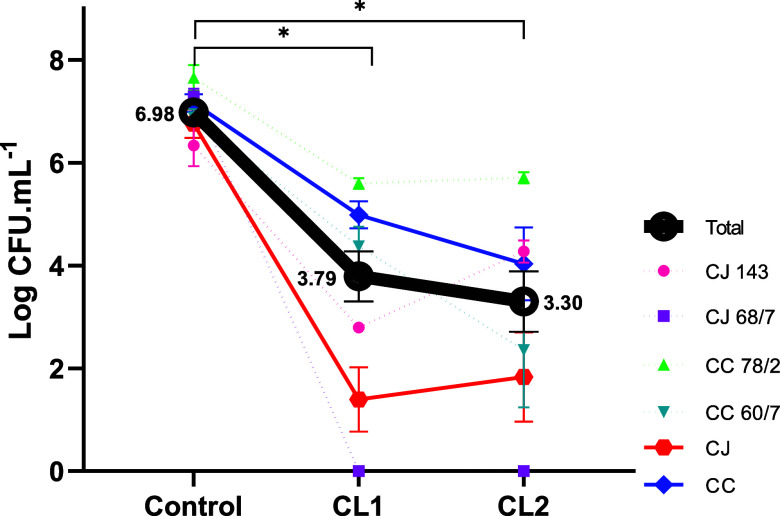
Decreased microbial enumeration of *Campylobacter jejuni* exposed to **CL1** and **CL2** (400 μg/mL) metal compounds. CJ 143 and 68/7: *C. jejuni* strains. CC 78/2 and 60/7: *C. coli* strains. *p < 0.0001 in Kruskal–Wallis test.

### 3.3 Impact of Copper­(II) Complexes CL1 and CL2 on the Biomass of *Campylobacter*

After treatment with the commercial standard sanitizer (APA [peracetic acid] 800 μg/mL) and the copper complexes, a significantly lower weight biomass intensity was observed in the case of CL2, compared to the control (*p* < 0.05). These reductions were 1.24 for *C. jejuni* and 1.25 for *C. coli* (Figure 3a,b).

**Figure 3:**
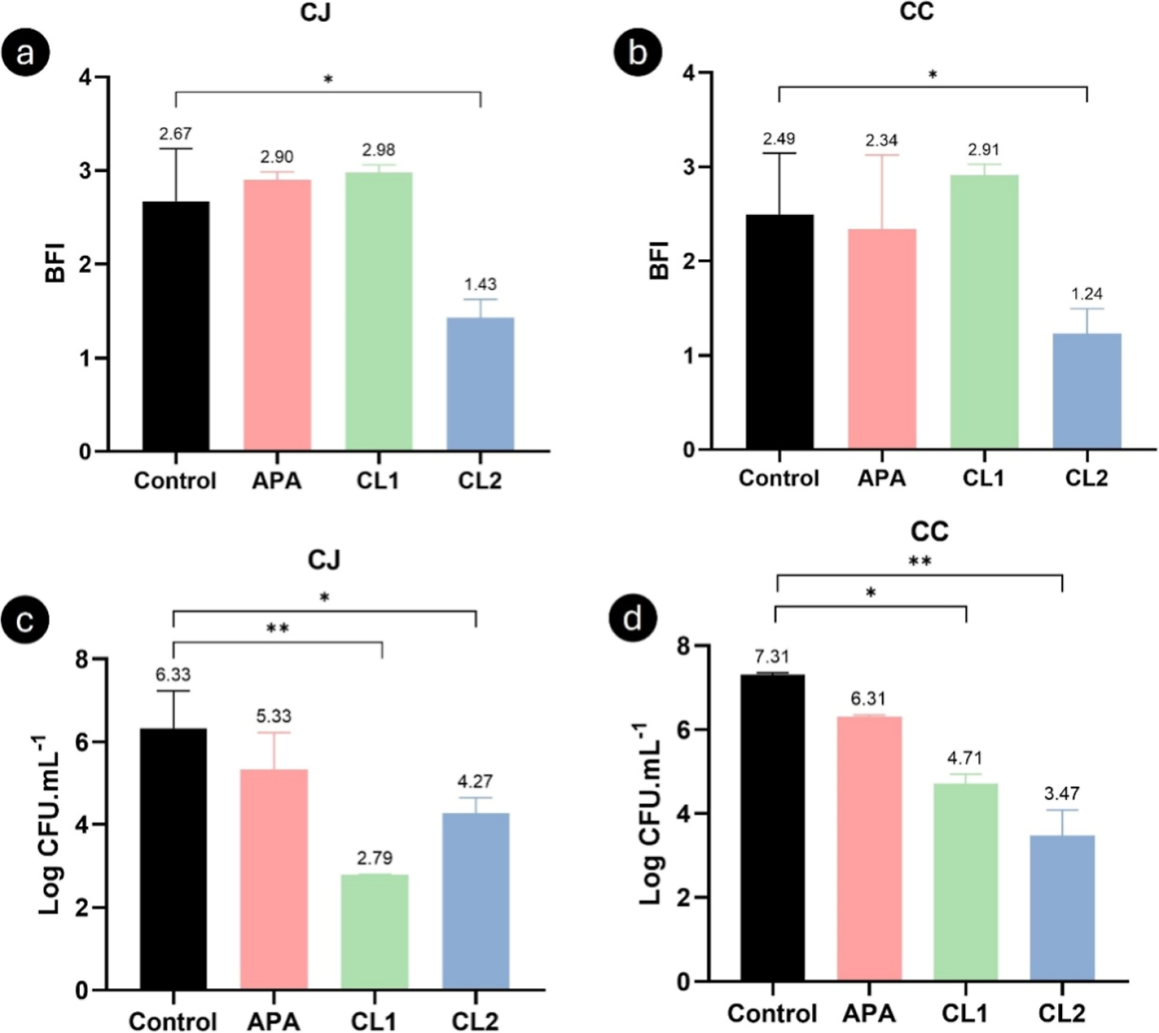
Discrimination of BFI of CJ (*C. jejuni*) (a) and CC (*C. coli*) (b) biofilms under contact with APA and copper complexes (**CL1** and **CL2**) and biofilm counts of CJ (c) and CC (d) (log CFU/mL) in the same conditions. *p < 0.05; **p < 0.001 using one-way ANOVA.

The reduction in sessile cell count was significant with both **CL1** and **CL2** (*p* < 0.05). Despite the maintenance of the extracellular matrix of the biofilm detected in Figure 3a,b, complex **CL1** demonstrated greater effectiveness in controlling *C. jejuni* in sessile form, with a reduction of 3.54 log cycles (*p* < 0.0001) (Figure 3c) and in *C. coli* of 2.60 (*p* < 0.05) (Figure 3d).

SEM analysis revealed modified biofilm biomass in the three treatments (APA, CL1, and CL2). The control group presented in Figure 4a, when organic matter is present, shows excessive biomass with a denser and more defined matrix. In Figure 4b, the biofilm treated with APA has a three-dimensional (3D) structure with a more developed (but less dense) matrix, slightly larger and spongy compared with that of the control. In the case of CL1 (Figure 4c), the biofilm presented a high bacterial mass but with reduced matrix and being more compact when compared to that generated by APA. CL2 (Figure 4d) exhibited the most dramatic changes with a visible breakdown and disruption of the matrix and loss of 3D structure, which was concurrent with the reduction in biomass detected by crystal violet assay (Figure 3a,b).

**Figure 4:**
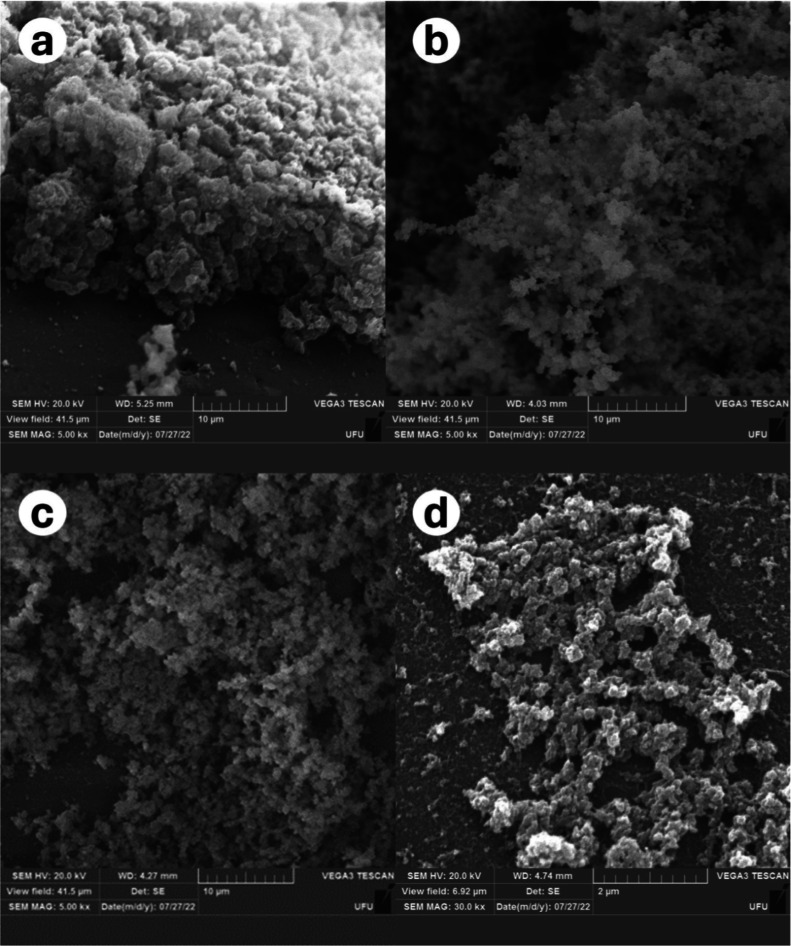
SEM images showing the ultrastructure of the biofilm architecture of *Campylobacter* *.* (a) Control group; (b) use of the sanitizer APA; (c) **CL1**; (d) **CL2**.

The atomic absorption test in biofilms (Figure 5) allowed the determination of the amount of copper that penetrated the sessile *Campylobacter* cells specific to each investigated complex and justified the discrepancies we identified in the counts after contact with **CL1** and **CL2** (Figure 4c,d). The greater activity of **CL1** in sessile *C. jejuni* is justified by the 2.18 times higher copper absorption compared to *C. coli* and 2.54 times higher copper absorption compared to **CL2**. The effect of **CL2** in *C. coli*, despite being greater in terms of the number of viable sessile cells (Figure 4d) and the amount of copper absorbed (Figure 5), did not differ significantly (*p* > 0.05) compared to *C. jejuni*.

**Figure 5:**
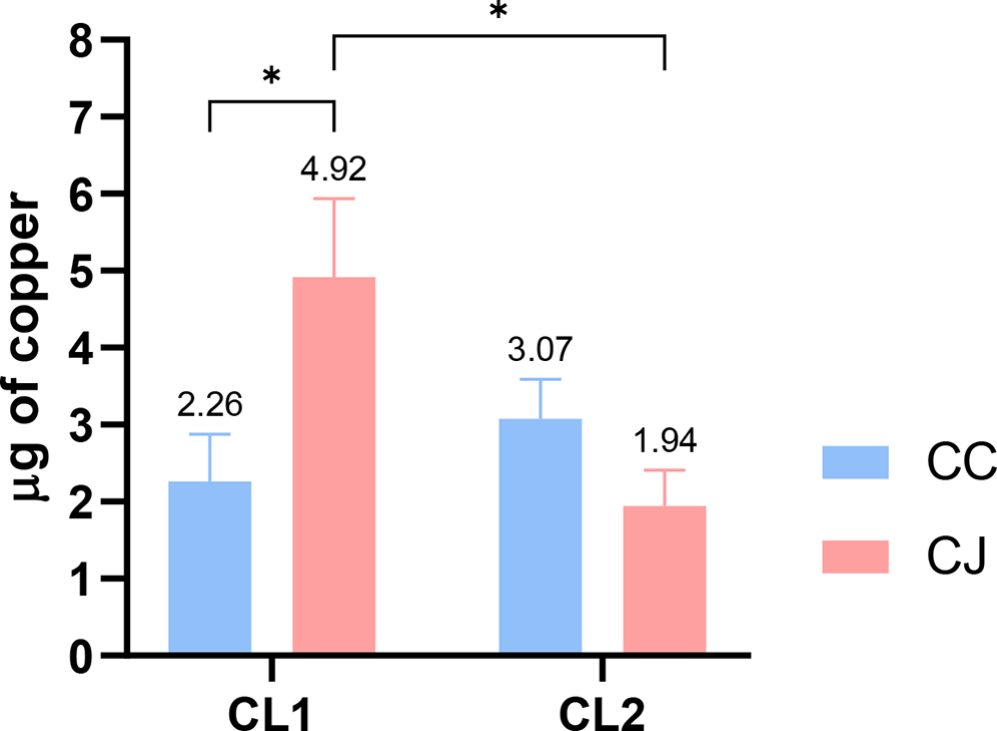
AAS in ppm of copper in CC (*C. coli*) and CJ (*C. jejuni*) sessile cells in contact with 800 μg/mL of CL1 and CL2. *p < 0,05 in *t* test.

## 4 Discussion

### 4.1 Synthesis and Spectroscopic Characterization of Complexes

The obtained results confirmed the identity and purity of the Clmp and memp ligands as well as the formation of the copper­(II) complexes **CL1** and **CL2**. The molar conductivity values, close to 285 S cm^2^ mol^–1^, suggest that both complexes act as 1:2 electrolytes, the PF_6_^–^ anions acting as counterions.^2,22^ The mass spectra corroborated the proposed structures, as the detected ions exhibited masses very close to the calculated values with insignificant errors.

The changes observed in the UV–vis spectra indicate electronic interactions between the metal and the ligands. For instance, the UV–vis spectra of the complexes showed the same bands with slight red or blue shifts due to coordination.^23,24^ A broad band near 614 nm is characteristic of d–d transitions in copper­(II) complexes with square-pyramidal geometry.^25^ The absence of significant changes in solution (acetonitrile) suggests that the geometry of the complex remains stable, indicating that this geometric stability may be a relevant factor for its chemical and biological properties.

The copper­(II) complexes were further analyzed by EPR spectroscopy as the environment of the central metal ion can significantly influence the biological properties of the complex. EPR spectra of complexes **CL1** and **CL2** exhibited very similar structural features, as expected, indicating a typical square-pyramidal environment around the copper ion, with similar spectroscopic parameters.

### 4.2 Anti-*Campylobacter* Activity

The differences in susceptibility between ATCC and wild-type strains can be attributed to genetic heterogeneity within the species. Wild-type strains, minimally subcultured, tend to exhibit greater phenotypic variability compared to the standardized strains from culture collections, which may influence their response to novel compounds.^26^ The resistant behavior of the CC78/2 strain was anticipated due to the presence of the copA gene in its chromosome (NCBI, SAMN38340616). This gene encodes the CopA protein, a copper transporter that aids in the homeostatic regulation of copper within the cell, preventing lethal accumulation and reducing copper’s cytotoxic effects.^27^

The results indicate that the anti-Campylobacter activity of the CL1 and CL2 complexes is directly linked to the ligand present in the structure. CL1, containing the Clmp ligand, demonstrated greater efficiency against *C. jejuni* ATCC than CL2, which possesses the memp ligand. We chose not to evaluate the free ligands in isolation, as the study’s focus was on the synergistic effect of metal–ligand coordination, which is the basis for the distinct biological potential of these complexes. The literature shows that coordination to the metal ion alters critical physicochemical properties, such as polarity and lipophilicity, thereby increasing the ability to permeate the bacterial membrane and enhancing the affinity for intracellular targets.^14,33^ Indeed, chelation with Cu­(II) can increase the compound’s lipophilicity, facilitating its passage across the bacterial cell wall and consequently intensifying its antimicrobial activity.

Regarding the greater efficiency observed for CL1, we believe this behavior is related not only to the electronic substitution on the imine ligand’s ring but also to stereoelectronic and charge distribution effects provided by the presence of the chlorine atom. According to previous studies, halogenated substituents, especially chlorine, increase the complex’s stability and its capacity for interaction with biomolecules via inductive and polarization effects.^26,34^ These factors may justify both the higher copper penetration (2.54 times greater) and the more pronounced logarithmic reduction observed for CL1 against *C. jejuni*. Overall, the logarithmic reduction demonstrates that both complexes are biologically active, triggering processes that enable pathogen control with an average bacterial population reduction of 99.9%.

In our study, the MIC values observed are similar to those described in another study where Schiff bases were synthesized through the condensation of 8-alkyl-2-hydroxy-tricyclo[7.3.1.02.7]-tridecan-13-one and 4-amino-2,3-dimethyl-1-phenyl-3-pyrazolin-5-one, alkyl: C2H5, *n*-C3H7, *i*-C3H7, C6H5; BS5: isonicotinic acid 2-(2-hydroxy-8-substituted-tricyclo[7.3.1.02.7]­tridec-13-ylidene)-hydrazones, alkyl: CH_3_, C_2_H_5_, *n*-C_3_H_7_, *i*-C_3_H_7_, and the antimicrobial and antibiofilm activities of the resulting complexes were tested in the strains of *S. aureus*, *E. coli*, *B. subtilis*, *P. aeruginosa*, and *Campylobacter albicans*. The authors noted that bacterial control ranged from 125 to 500 μg/mL and that the ligands exhibited satisfactory antibiofilm activity, likely due to more efficient penetration of ligand molecules into the protective biofilm matrix.^28^

Similarly, other studies have investigated binary and ternary Cu­(II) complexes containing l-arginine, [CuCl­(l-Arg)­(phen)]­Cl.2H_2_O (phen = 1,10-phenanthroline) (**1**), and [Cu­(l-Arg)_2_(H_2_O)]­C_2_O_4_.6H_2_O (**2**), for their antitumor activity against lung cancer and hepatocellular carcinoma cells, as well as antimicrobial activity against ten microorganisms in planktonic form. For all strains, the MIC values for complexes 1 and 2 were ≤15 μM, although complex 2 showed a higher susceptibility than complex 1. Additionally, Gram-negative strains *P. aeruginosa*, *E. coli*, *S. Typhimurium*, and *S. flexneri* showed higher resistance to the complexes than Gram-positive strains, with MIC values of 12.5 μM for *P. aeruginosa* and MIC values below 10 μM for the others.^29^

The effects of copper­(I) complex [Cu­(NN1)_2_]­ClO_4_ with a coumarin ligand on bacterial growth reduction and inhibition of biofilm formation by *Vibrio harveyi* were reported in a 2021 study. Serial dilutions of the copper­(I) complex, from 1024 to 2 μg/mL, demonstrated lower bacterial growth compared to other treatments, with the lowest optical density values close to zero at the highest levels (256/512/1024 μg/mL). In terms of biofilm reduction (0–48 h), all tested concentrations of the copper­(I) complex were significant (*p* < 0.05). At 12.6 μg/mL, an 80% reduction in biofilm biomass was observed compared to the untreated control group. The antibiofilm effect decreased at 6.3 μg/mL due to the lower concentration of the complex. The authors concluded that less copper was required to achieve the same antibacterial effects observed in control treatments with coumarin and copper salt.^30^

### 4.3 Impact of Copper­(II) Complexes CL1 and CL2 on the Biomass of *Campylobacter*

The SEM visualization reveals distinct ultrastructural differences of Campylobacter biofilm formation in the control group (a) as well as following treatment with the sanitizer APA and the copper­(II) complexes **CL1** and **CL2**. These differences are particularly evident in the morphology and density of the biofilm matrix, with a more pronounced disruption of biofilm integrity observed in the presence of **CL1** and **CL2**, compared to that in the control and APA-treated groups. The biofilms treated with **CL1** and **CL2** exhibit significant thinning and partial detachment from the surface, suggesting the complexes’ potential in inhibiting biofilm formation and disrupting existing biofilms. In contrast, the control group showed a dense and compact biofilm, and the APA-treated biofilm, although less dense than the control, did not exhibit the same extent of disruption as that seen with **CL1** and **CL2**.

In general, available data indicate that several Cu­(II) complexes containing imine bases exhibit strong antibiofilm activity against a wide range of Gram-positive and Gram-negative bacteria.^31^ However, this study is the first to investigate and confirm the antimicrobial and antibiofilm effects of these complexes on *Campylobacter*. The results suggest that the effectiveness of these complexes may be attributed to their ability to penetrate the biofilm and disrupt the protective matrix, which is critical for the persistence and resistance of *Campylobacter* in both environmental and clinical settings.

The atomic absorption test performed on biofilms enabled the quantification of copper penetration into sessile *Campylobacter* cells, allowing us to assess the amount of copper specific to each investigated complex. These data explain the discrepancies observed in bacterial counts following exposure to **CL1** and **CL2**. The penetrability of the cupric ion (Cu­(II)) was found to be higher compared to the cuprous ion (Cu­(I)), as Cu­(II) is biologically inert and better recognized by bacterial systems, making it more readily transported into bacterial cells.^32^ This enhanced penetrability facilitates the targeting and control of both free-swimming and sessile forms of *Campylobacter*, suggesting that Cu­(II)-based complexes may offer a promising strategy for controlling biofilm-related infections caused by *Campylobacter*.

## 5 Conclusions

The microbiological effects provided by both copper complexes with imine ligands are very promising in both life forms of *Campylobacter* and appear as an alternative for the control of this pathogen of relevant importance for public health.

## Supplementary Material



## Notes

^1^H NMR spectra of Clmp and memp ligands; mass spectra of copper complexes CL1 and CL2; UV–Vis spectra of CL1 and CL2 and their respective ligands in acetonitrile; diffuse reflectance spectra of CL1 and CL2; IR spectra of CL1 and CL2 and their respective ligands; and EPR spectra of CL1 and CL2 in solid state and in DMSO solution (PDF)
