# Supplementary material for: Anti-Campylobacter Activity of Ternary Copper(II) Complexes with Imine Ligands and 4′-(4-Methylphenyl)-2,2′:6′,2″-Terpyridine
Source: ACS Omega. 2026 Jan 23;11(4):5042–50. doi: 10.1021/acsomega.5c06248 (PMC12878296; doi:10.1021/acsomega.5c06248)

Supporting Information

Anti-*Campylobacter* activity of ternary copper(II) complexes with imine ligands and 4'-(4-methylphenyl)-2,2':6',2''-terpyridine

Micaela G. Takeuchi[a], Ana Laura M. Ferreira[b*], Luana M. S. Ramos[c], Jéssica Laura M. Peixoto[b], Mariana C. Chueiri[b], Carolyne F. Dumont[b], Gabriella R. A. Ferreira[b], Diogo M. de Jesus[c], Thiago dos S. Ramos[c], André L. Bogado[c], Gabriele de M. Pereira[f], Marcelo C. Portes[d], Pedro P. Corbi[f], Ana Maria da C. Ferreira[d], Daise A. Rossi[b], Wendell Guerra[c*], Roberta T. de Melo[b*]

[a]Federal Institute of Rio Grande do Sul, Farroupilha Campus, 95174-274, Farroupilha, Rio Grande do Sul, Brazil

[b]Molecular Epidemiology Laboratory, Federal University of Uberlândia, Umuarama Campus, 38400-902, Uberlândia, Minas Gerais, Brazil

[c]Institute of Chemistry, Federal University of Uberlândia, Santa Mônica Campus, 38400-902, Uberlândia, Minas Gerais, Brazil

[d]Department of Fundamental Chemistry, Institute of Chemistry, University of São Paulo, 05508-000, São Paulo, São Paulo, Brazil.

[e]Department of Chemistry, Institute of Exact and Natural Sciences of Pontal, Federal University of Uberlândia, 38304-402, Ituiutaba, Minas Gerais, Brazil

[f]Institute of Chemistry, State University of Campinas – UNICAMP, 13083-862, Campinas, São Paulo, Brazil

ana.ferreira2@ufu.br, wendell.guerra@ufu.br, roberta.tmelo@ufu.br

Figure S1. ^{1}H NMR spectrum of the ligand Clmp ($CDCl_3$, 400 MHz).

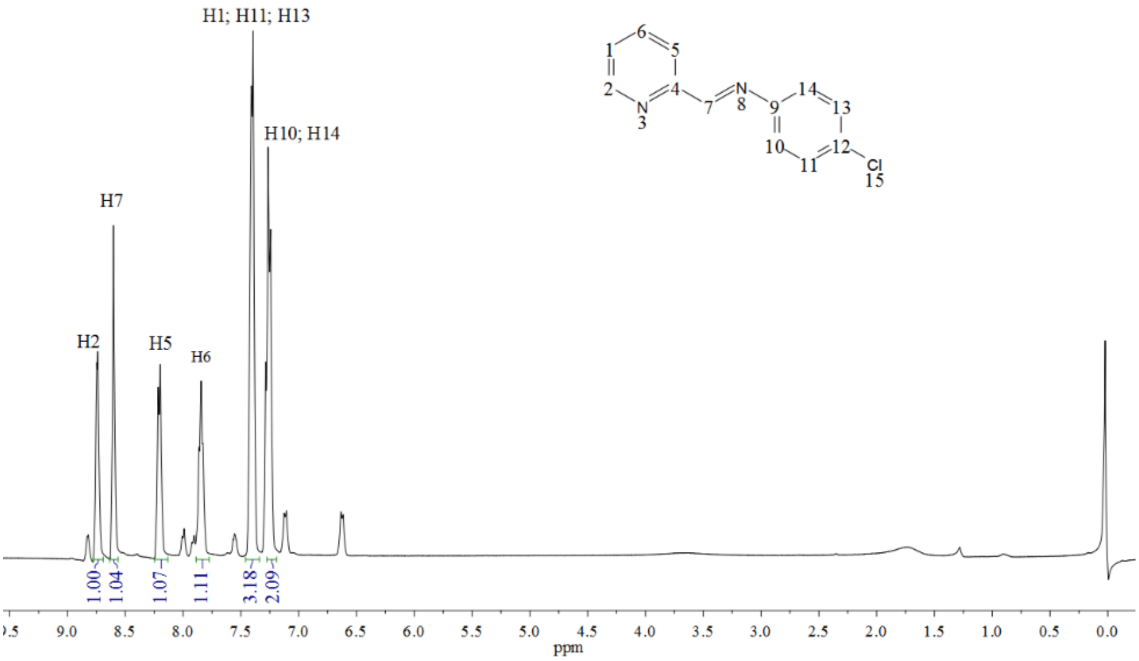

Figure S2. ^{1}H NMR spectrum of the ligand memp ($CDCl_3$, 400 MHz).

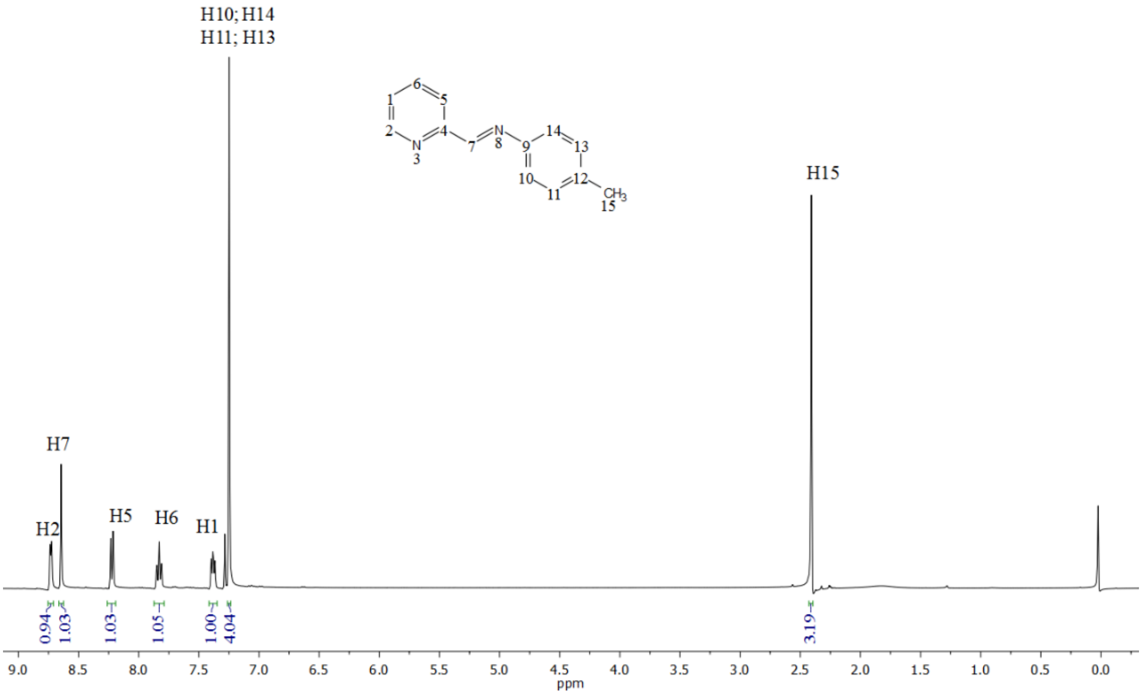

Figure S3. Mass spectrum for the **CL1** complex. (A) Full spectrum, *m/z* 100-1500. (B) Expanded spectrum and (C) calculated isotopic pattern for the $[C_{34}H_{26}ClCuN_5]^{+2}$ molecular ion.

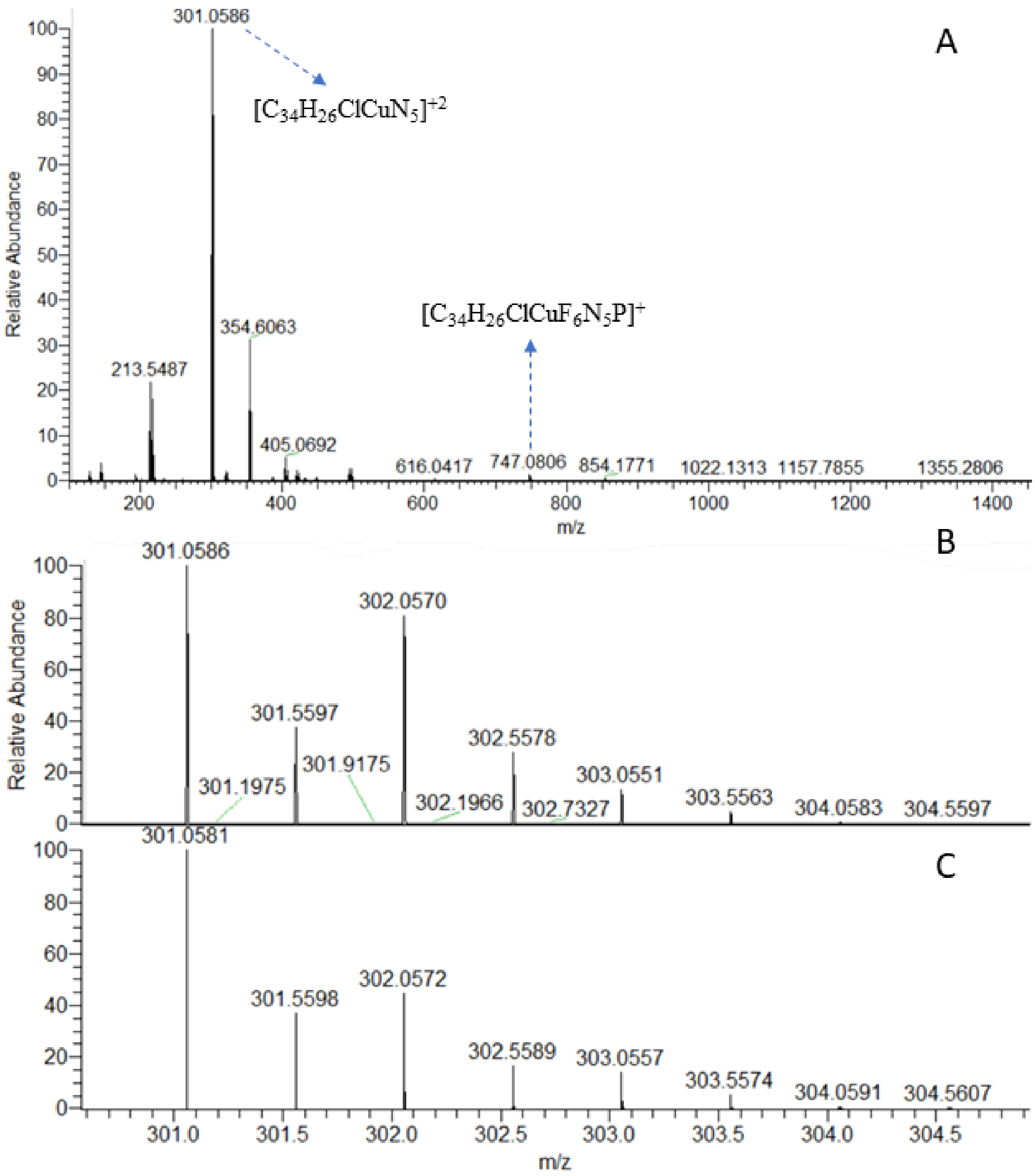

Figure S4. Mass spectrum for the **CL2** complex. (A) Full spectrum, *m/z* 100-1500. (B) Expanded spectrum and (C) calculated isotopic pattern for the $[C_{35}H_{29}CuN_5]^{+2}$ molecular ion.

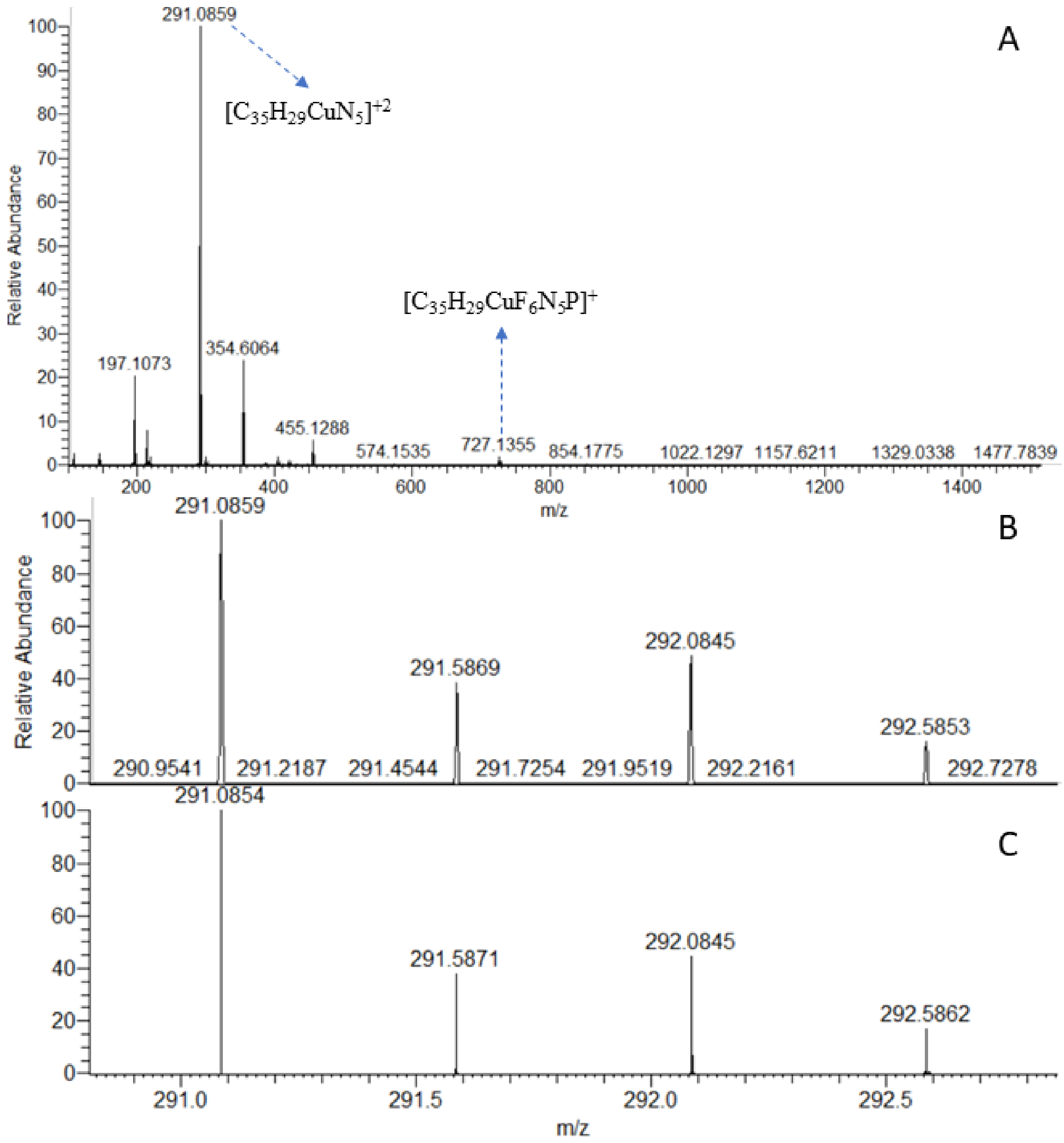

Figure S5. UV-Vis spectrum of **CL1** and their respective ligands in acetonitrile.

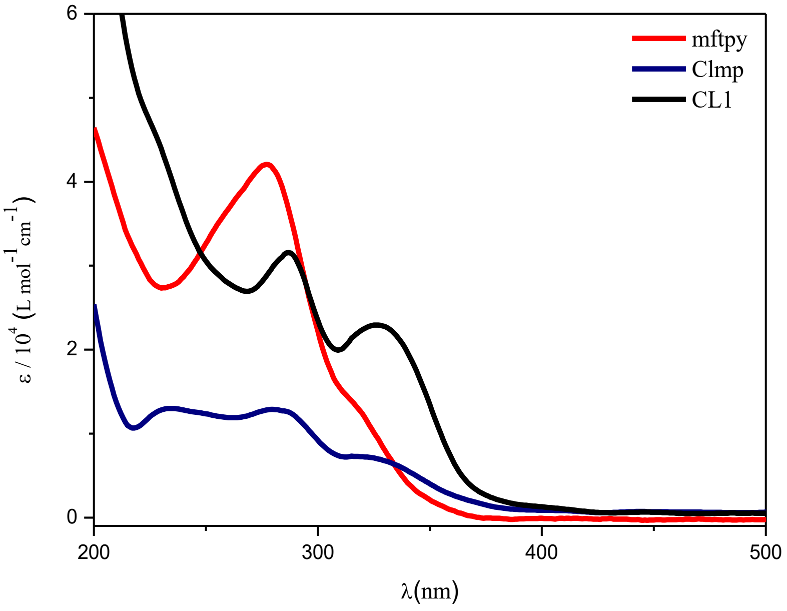

Figure S6. UV-Vis spectrum of **CL2** and their respective ligands in acetonitrile.

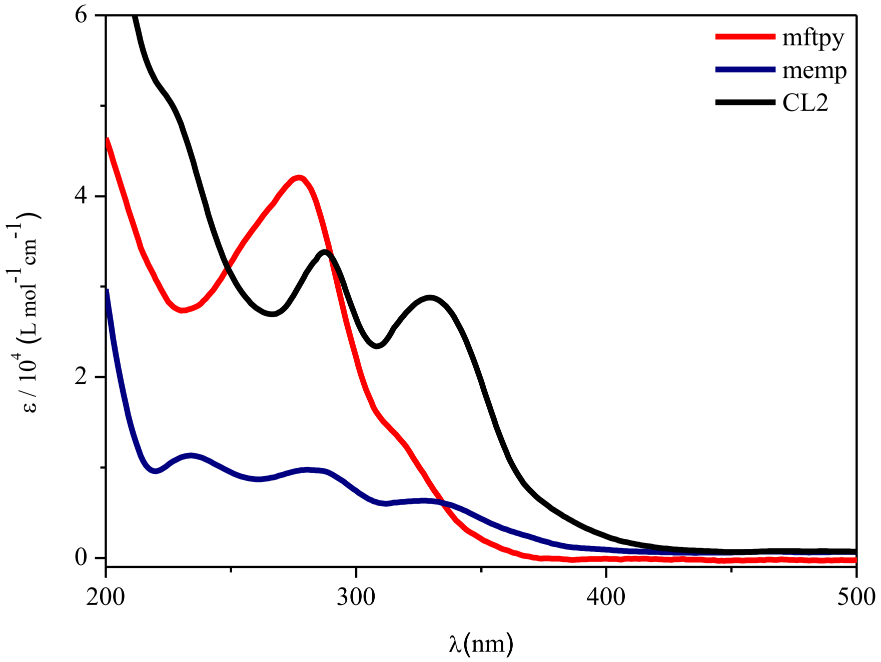

Figure S7. Diffuse reflectance spectra of **CL1** and **CL2**.

CL1

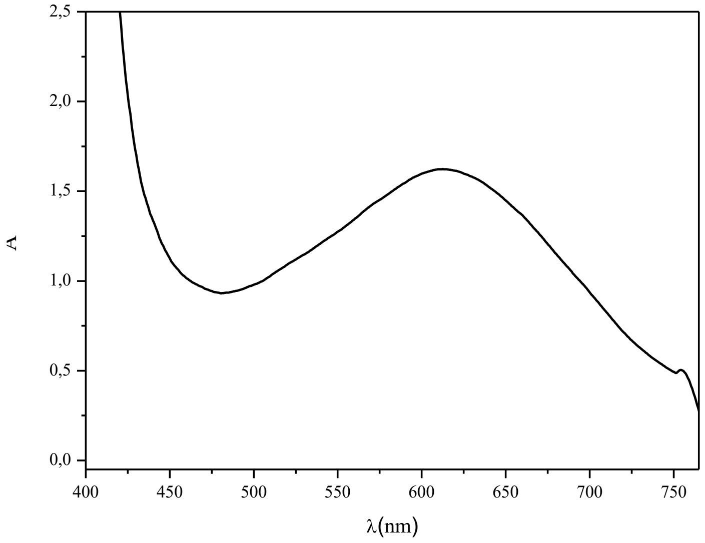

CL2

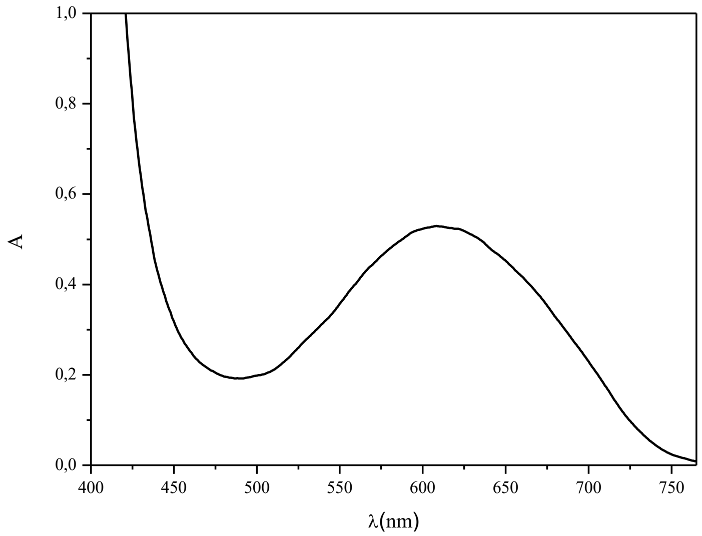

Figure S8. IR spectra of **CL1** and their respective ligands.

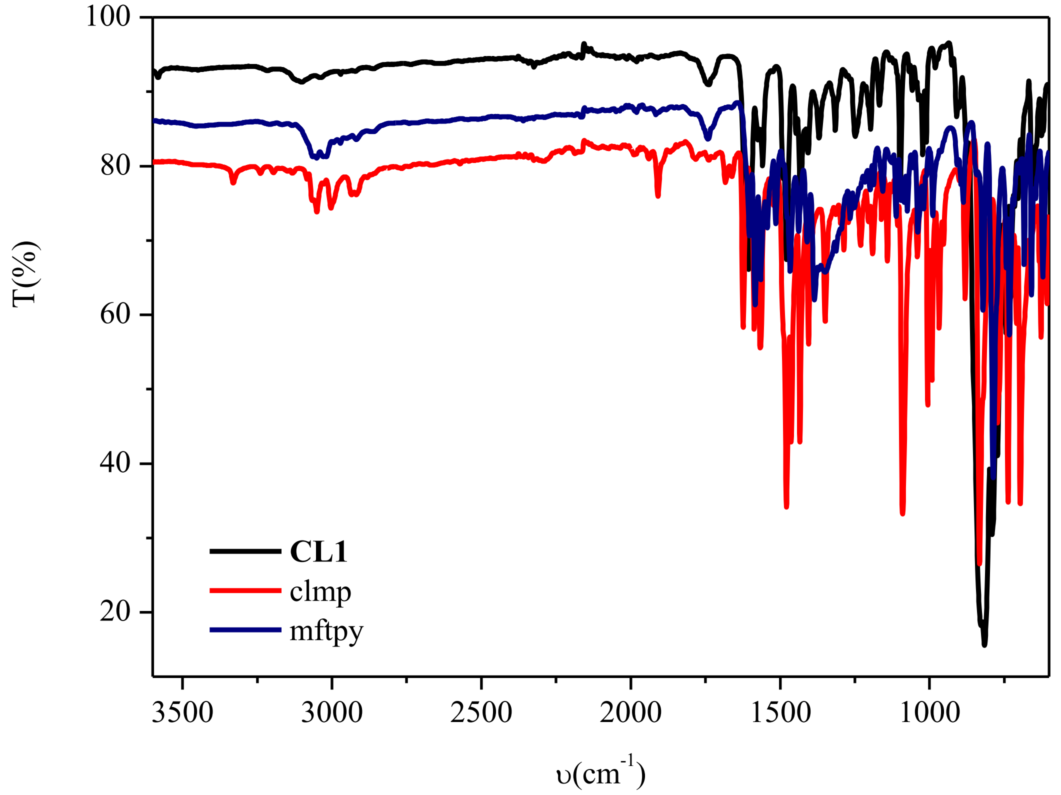

Figure S9. IR spectra of **CL2** and their respective ligands.

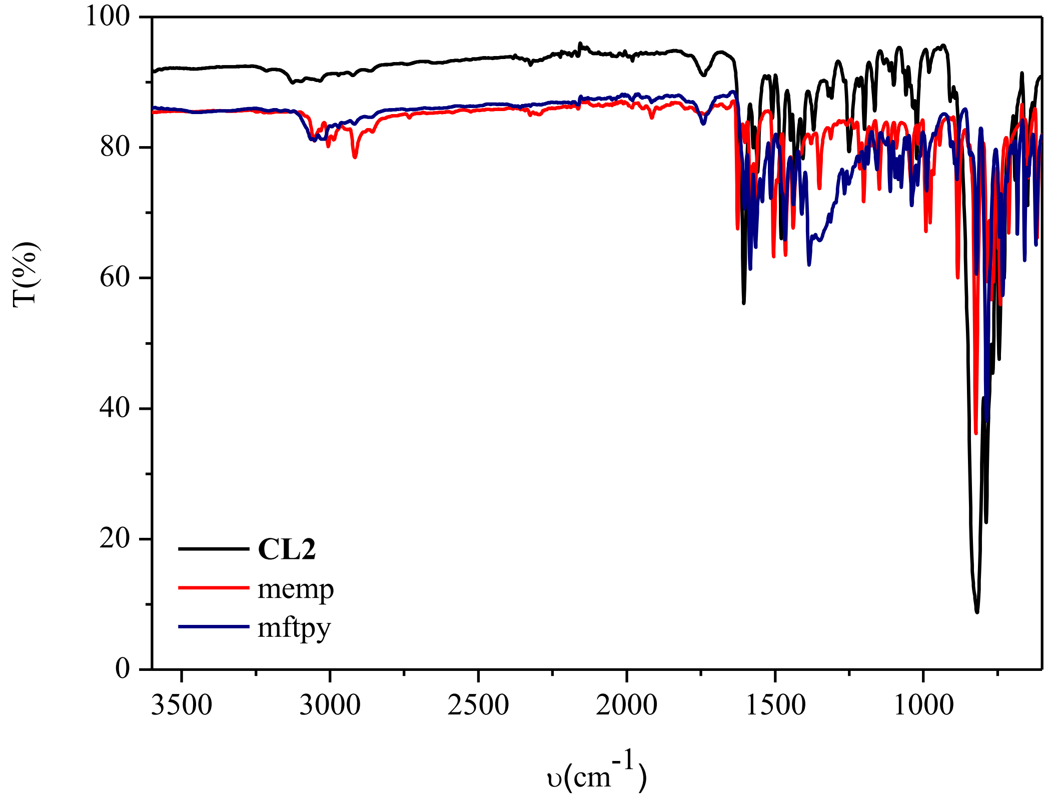

Figure S10. EPR spectra of complex **CL1** in solid-state (left) and in DMSO solution (right) at room temperature. Blue arrow indicates hyperfine structure due to N coordinating atoms.

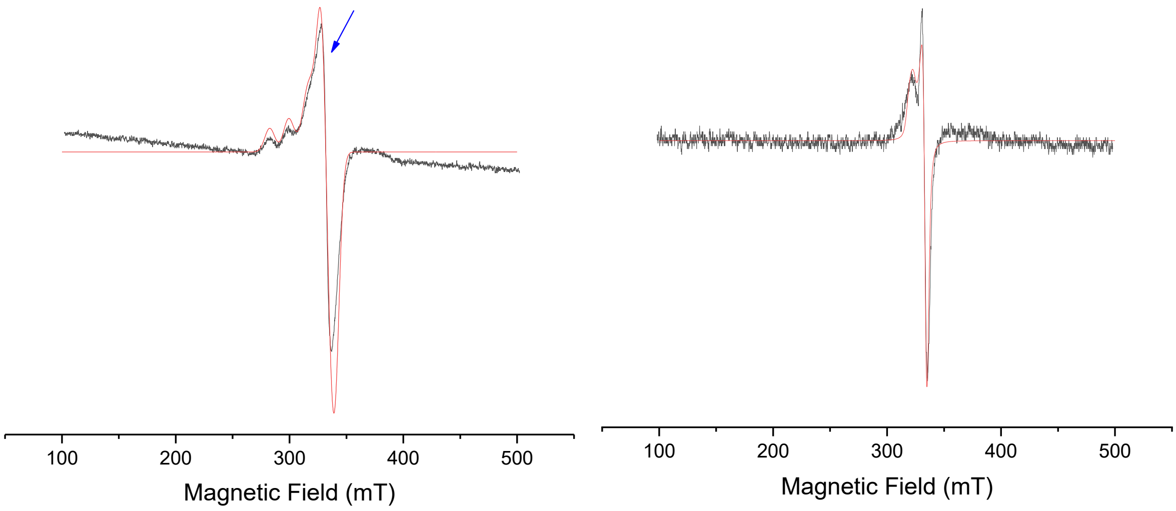

Figure S11. EPR spectra of complex **CL2** in solid-state (left) and in DMSO solution (right) at room temperature. Blue arrow indicates hyperfine structure due to N coordinating atoms.

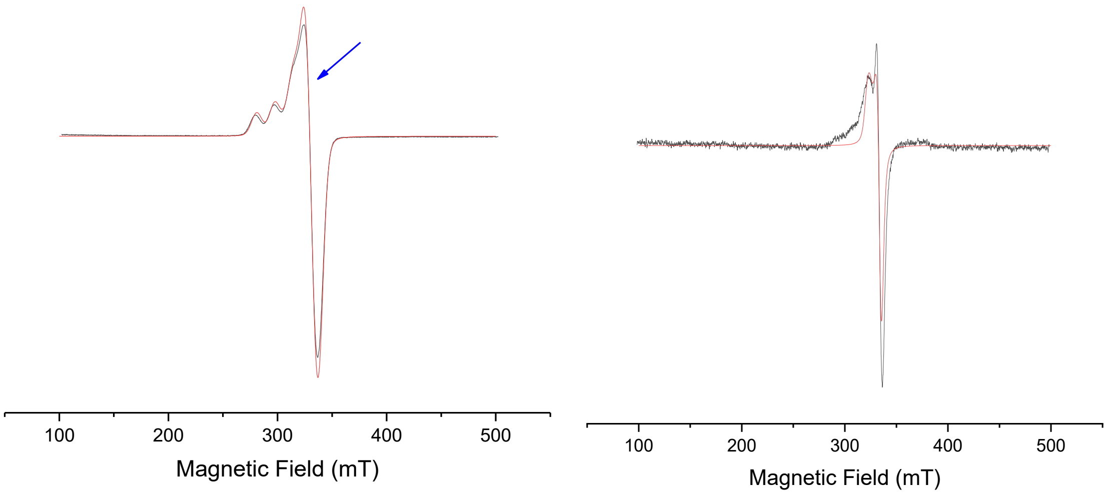

Supplement: Supplementary file 1 [file ao5c06248_si_001.pdf]
